# Recent Advances in Clinical Diagnosis and Pharmacotherapy Options of Membranous Nephropathy

**DOI:** 10.3389/fphar.2022.907108

**Published:** 2022-05-26

**Authors:** Yan-Ni Wang, Hao-Yu Feng, Xin Nie, Ya-Mei Zhang, Liang Zou, Xia Li, Xiao-Yong Yu, Ying-Yong Zhao

**Affiliations:** ^1^ Faculty of Life Science & Medicine, Northwest University, Xi’an, China; ^2^ Key Disciplines of Clinical Pharmacy, Clinical Genetics Laboratory, Affiliated Hospital and Clinical Medical College of Chengdu University, Chengdu, China; ^3^ School of Food and Bioengineering, Chengdu University, Chengdu, China; ^4^ Department of General Practice, Xi’an International Medical Center Hospital, Northwest University, Xi’an, China; ^5^ Department of Nephrology, Shaanxi Traditional Chinese Medicine Hospital, Xi’an, China

**Keywords:** chronic kidney disease, idiopathic membranous nephropathy, membranous nephropathy, traditional Chinese medicine, *Astragalus membranaceus*, *Tripterygium wilfordii*, Astragaloside IV, Shenqi particle

## Abstract

Membranous nephropathy (MN) is the most common cause of nephrotic syndrome among adults, which is the leading glomerular disease that recurs after kidney transplantation. Treatment for MN remained controversial and challenging, partly owing to absence of sensitive and specific biomarkers and effective therapy for prediction and diagnosis of disease activity. MN starts with the formation and deposition of circulating immune complexes on the outer area in the glomerular basement membrane, leading to complement activation. The identification of autoantibodies against the phospholipase A_2_ receptor (PLA_2_R) and thrombospondin type-1 domain-containing protein 7A (THSD7A) antigens illuminated a distinct pathophysiological rationale for MN treatments. Nowadays, detection of serum anti-PLA_2_R antibodies and deposited glomerular PLA_2_R antigen can be routinely applied to MN. Anti-PLA_2_R antibodies exhibited much high specificity and sensitivity. Measurement of PLA_2_R in immune complex deposition allows for the diagnosis of PLA_2_R-associated MN in patients with renal biopsies. In the review, we critically summarized newer diagnosis biomarkers including PLA_2_R and THSD7A tests and novel promising therapies by using traditional Chinese medicines such as *Astragalus membranaceus*, *Tripterygium wilfordii*, and Astragaloside IV for the treatment of MN patients. We also described unresolved questions and future challenges to reveal the diagnosis and treatments of MN. These unprecedented breakthroughs were quickly translated to clinical diagnosis and management. Considerable advances of detection methods played a critical role in diagnosis and monitoring of treatment.

## 1 Introduction

Membranous nephropathy (MN) is one of the most common causes of formation of nephrotic syndrome in adults, accounting for 30% incidence of patients (1.7/100000/year), with a 67% male preponderance and a high incidence in humans aged 30–50 years (Bally et al., 2016). It is unwonted in children (Liu et al., 2020a; Tamura, 2021). MN mainly affects renal glomerulus, particularly podocytes in glomerulus, indicating that podocytes play a critical role in regulating renal permeability to various molecules including proteins (Ronco and Debiec, 2020). In healthy individuals, albumin and macromolecule proteins are not filtered, while in a milieu called nephrotic syndrome, many proteins are leaked and excreted through urine, leading to a reduction in serum albumin and generalized edema development (Ronco and Debiec, 2020; Medina Rangel et al., 2021).

**FIGURE 1:**
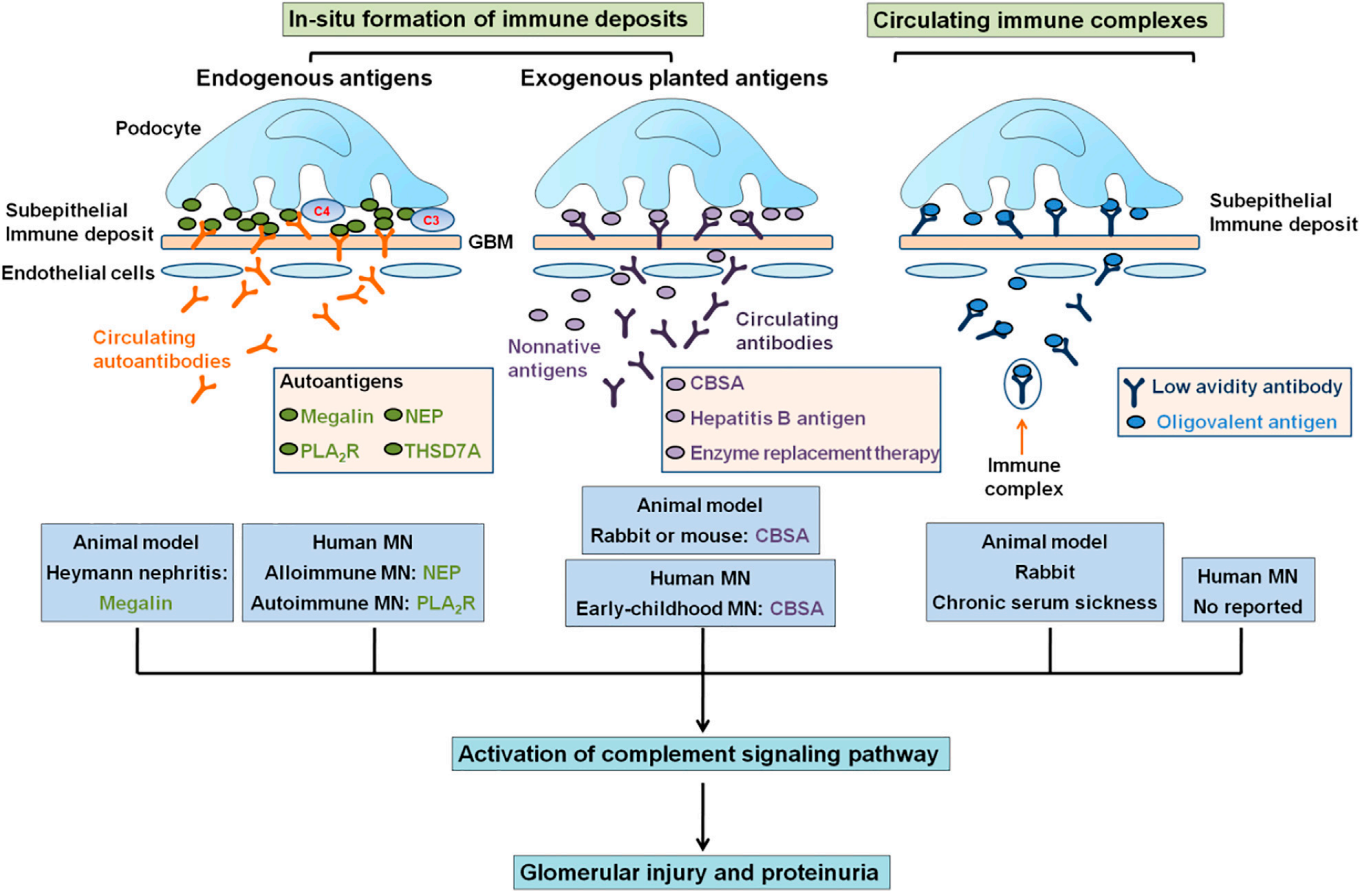
Mechanisms of formation of subepithelial immune complex deposits. The depositions of subepithelial immune complexes might form either *in situ* immune complexes or circulating immune complexes that contained circulating antibodies binding to endogenous antigens (megalin, NEP, PLA_2_R, and THSD7A) in podocytes or to exogenous antigens (CBSA, hepatitis B antigen, and enzyme replacement therapy) planted in GBM. In total, four endogenous antigenic targets have been identified, including megalin in Heymann nephritis, NEP in alloimmune neonatal MN, and PLA_2_R and THSD7A in IMN. In total, four circulating exogenous antibodies can target surface-exposed intrinsic podocyte antigens to cause capping and shedding of *in situ* antigen–antibody complex into underlying GBM. CBSA, enzyme from enzyme replacement therapy, and hepatitis B antigen may traverse GBM and bind under podocyte as planted antigens and also as target for circulating antibodies. In addition, circulating immune complexes deposited in the subepithelial region.

This condition can be “primary” or “idiopathic” for patients that did not present disease association (70–80% of patients), or for patients that present disease association, such as infections, lupus erythematosus, malignancy, or drug toxicity (Moroni and Ponticelli, 2020; Cravedi et al., 2019). Exogenous antigens may pass *via* the glomerular basement membrane (GBM), become planted under the podocyte surface layer, and following the combination with circulating antibodies (Cravedi et al., 2019) (Figure 1). Circulating immune complexes separate and reform in the subepithelial space (Moroni and Ponticelli, 2020). Idiopathic membranous nephropathy (IMN) is a kidney-specific non-inflammatory autoimmune disease, and circulating autoantibodies bind to autoantigens on the podocyte surface layer (Moszczuk et al., 2021). About 40% of IMN patients could suffer spontaneous remission. However, the rest of 30% showed a poor outcome to immunosuppressive treatment and finally reached end-stage renal disease treated by dialysis and transplantation (Tesar and Hruskova, 2021; Passerini et al., 2019; Xipell et al., 2018; Molina Andújar et al., 2022; da Silva et al., 2018), which were two leading therapies for patients with end-stage renal disease (Zhang et al., 2020a; Sawhney and Gill, 2020; Wu et al., 2021a; Bacharaki et al., 2021; Chang et al., 2021; Chuengsaman et al., 2021; Gambino et al., 2021). Approximately, 40% patients accept kidney grafts that lead to recurrence, and about 45% patients lose the graft (Passerini et al., 2019; Robson and Kitching, 2021; Uffing et al., 2021). Treatment with costly drugs and potential adverse effects of drugs remain challenging (Gauckler et al., 2021). The most important points of precision therapy are the discovery of the accurate etiology and pathogenesis in IMN.

**FIGURE 2:**
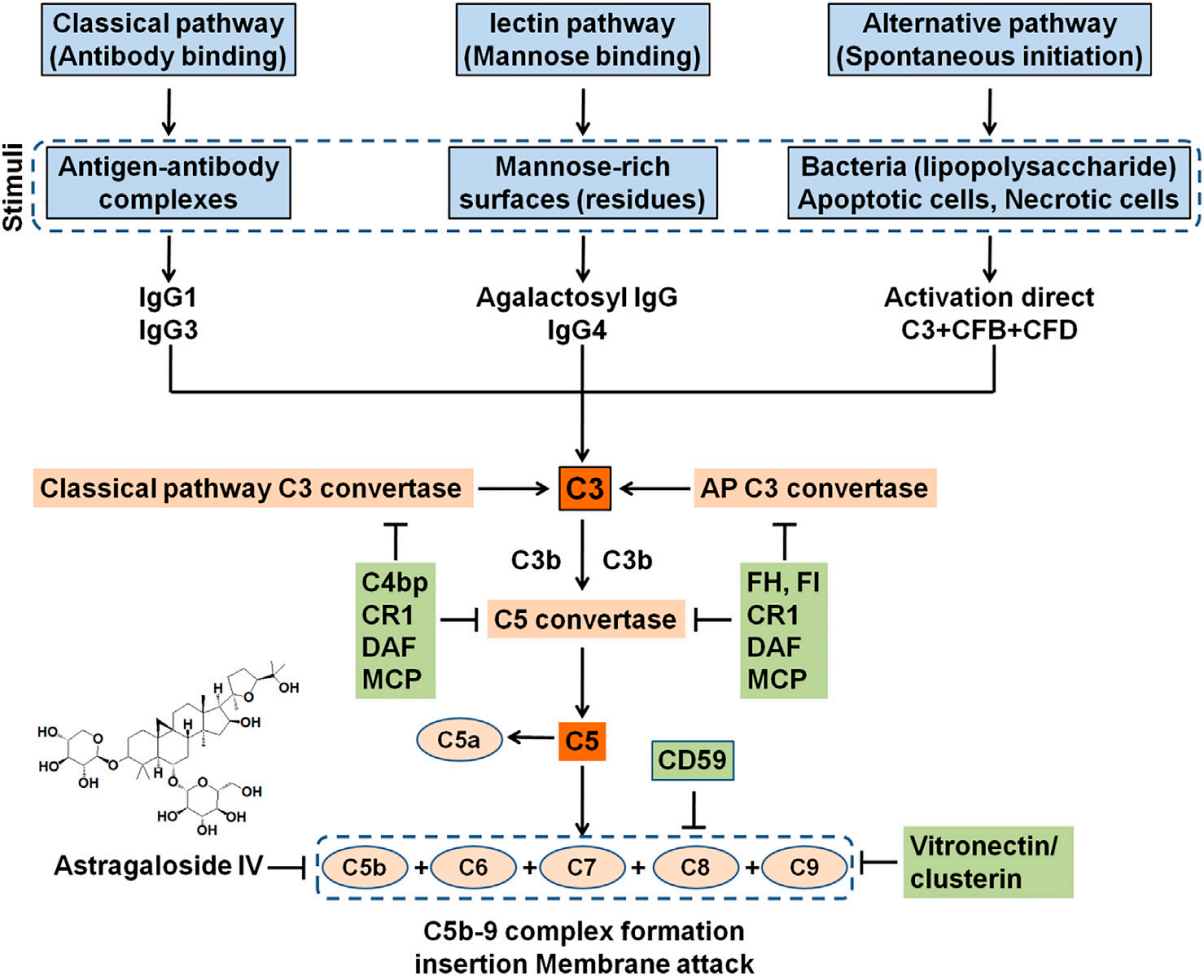
Activation of complement system-induced formation of MAC (C5b-9). Complement could be activated by three pathways including classical, lectin, and alternative signaling pathways, which came together toward production of C5 convertase and formation of C5b that bound to C6, C7, C8, and multiple C9 to generate the MAC. This process could be mostly inhibited by circulating inhibitors, podocyte-positive regulatory proteins, and natural products such as Astragaloside IV. However, this inhibitory effect might be devastated at high levels of complement. MAC could insert into the podocyte membrane, even in sublytic quantity, evoked cell injury under a lack of inflammation condition.

The feature of IMN is immune complex deposition along the subepithelial region of GBM, which leads to a membrane-like thickening of GBM (Liu et al., 2020b; Gu et al., 2021) (Figure 1). The immune complexes are composed of several components, such as IgG4, antigens that are eluded, and membrane attack complex, which is formed by complement components to produce C5b–9 (Gu et al., 2021; Hu et al., 2021) (Figure 2). IgG4 is the most main IgG subclass deposited in IMN, although altered IgG1 is also involved in the deposited immune complexes, IgG1-3 exceeds IgG4 deposition in secondary MN patients (Lönnbro-Widgren et al., 2015; Xu et al., 2020; Ronco et al., 2021). Subepithelial immune complex formation deposits and activation of complement are directly associated with functional lesion of the glomerular capillary wall, which leads to urine protein in absence of inflammatory cells (Xu et al., 2020; Gu et al., 2021) (Figure 2).

Based on the natural IMN history, IMN has a classic rule of thirds including one-third patients with spontaneous remission, one-third patients with sustaining proteinuria, and one-third patients with progression of kidney failure (McQuarrie et al., 2012). Although spontaneous remission is a common feature of IMN, IMN causes end-stage renal disease in nearly 40% of patients after 10 years (Ronco et al., 2021). Interventions remain controversial and challenging owing to adverse effect using immunosuppressive treatment, and, apart from proteinuria, few sensitive and reliable biomarkers are used for the prediction of disease activity and outcome because of the fact that antigens targeted by antibodies remain enigmatic (Moszczuk et al., 2021; Tesar and Hruskova, 2021). Taken together, an essential improvement of IMN diagnosis, monitoring, treatment, and prognosis is the elucidation of pathogenic mechanisms.

In the past several decades, important advances have been achieved in the illumination of the mechanisms of molecular pathogenesis of human MN (Gu et al., 2021; Xu et al., 2020). In addition, several latest publications have shown that MN was associated with the dysbiosis of gut microbiota and dysregulation of long non-coding RNAs (Zhang et al., 2020b; Dong et al., 2020; Luan et al., 2022; Jin et al., 2019). These advances were inspired by using animal models of IMN, including Heymann nephritis and cationic bovine serum albumin (CBSA)–induced MN (Jiang et al., 2020). The model of Heymann nephritis put forward the concept that a podocyte antigen, namely, megalin, was a target of antibody-forming *in situ* immune complexes, whereas CBSA-induced MN first reflected the case of planted antigen (Figure 1). Jones (1957) first reported MN as a specific disease entity. Subsequently, the recognition that autoimmune response to antigen-induced MN was first reported in an animal model in 1959. The rats were injected by using extracts from proximal tubular cells that mediated immune complex deposition on the subepithelial capillary wall region in the glomerular that were similar to those found in MN patients, strongly indicated the possibility of an immune-induced pathogenesis mechanism (Heymann et al., 1959). These immunocomplexes included IgG antibody–targeting megalin, which was expressed on both rat podocytes and tubuli (Kerjaschki and Farquhar, 1982), but not on podocytes in human. In humans, progress in MN started in 2002 with the discovery of target antigen, namely, neutral endopeptidase (NEP, also known as neprilysin) as a targeting antigen in baby of a woman with NEP deficiency (Debiec et al., 2002). Anti-NEP alloantibodies was generated by the mother-passed placenta and bound to NEP expressed in fetal podocytes (Debiec et al., 2002), which showed the role of autoantibody in human MN pathogenesis. This result showed the proof of concept that podocyte antigens were associated with human MN, as is the paradigm for megalin in rats, and laid the foundation for the identification of a novel causal antigen M-type phospholipase A_2_ receptor (PLA_2_R), which is the first podocyte autoantigen identified in human IMN in 2009 (Beck et al., 2009). In 2014, this was followed by the discovery of a second antigen, namely, thrombospondin type-1 domain-containing 7A (THSD7A) in human IMN (Tomas et al., 2014). PLA_2_R-related and THSD7A-related MN accounted for about 70% and 1–5% of IMN patients, respectively (Zhang et al., 2021a). A genome-wide association study indicated that single nucleotide polymorphisms in the *PLA*_*2*_ *R* gene were closely related to IMN, which again revealed the involvement of this antigen by using an untargeted genetic approach (Xu et al., 2020; Yoshikawa and Asaba, 2020). Other antigens including aldose reductase, superoxide dismutase-2, and α-enolase were also found in human, although their function remains to be established because they were not detected on normal podocyte surfaces (Prunotto et al., 2010). In addition, endogenous podocyte antigens and exogenous antigens including CBSA were also involved in patients with early-childhood MN (Ayalon and Beck, 2015; Jiang et al., 2020). Taken together, these studies exhibited a new era for the diagnosis, monitoring, and prognosis of MN from early infancy to adulthood. In this article, we summarize the traditional diagnostic method for MN and review recent improvements in diagnostics and the treatment of MN.

## 2 Diagnosis of Membranous Nephropathy

### 2.1 Traditional Diagnosis Approach

On the patients with nephrotic syndrome, after diagnosis for secondary causes, a renal biopsy was subsequently performed, which was a gold standard for MN diagnosis. The membranous summarized microscopic characteristic of capillary wall thickening in glomeruli, which led to subepithelial IgG accumulation. Immune complex depositions produced a spiked appearance and formed granular lines. Electron microscopy analysis further confirmed electron-dense subepithelial immune complexes, which were often accompanied by foot process effacement in podocyte. If the diagnosis was established, Kidney Disease: Improving Global Outcomes advised a 6-month observation, as there are many patients of spontaneous remission of MN. However, it takes more than a year to reach spontaneous remission.

### 2.2 Newer Diagnosis Approach

Renal biopsy is a gold standard in the analysis and detection of the pattern of MN damage. However, standard light and electron microscopic results could not reflect nature of MN (Xie et al., 2020). The distinction between IMN and secondary MN is the main challenge in the diagnosis of MN, in particular malignancy-related MN in patients. It is a common practice to exclude secondary causes of the lesion of MN on the basis of physical examination, pathological analysis, and laboratory examination. An assessment of the immunopathologic features from the biopsy specimen may get discriminative information (Ronco and Debiec, 2015). A sole subepithelial location of the immune complex deposits is typical characteristics of IMN. The deposition of C1q was hardly observed in IMN but could be found in other secondary causes, especially in systemic lupus erythematosus. IgG subclass staining could help to classify MN. The deposits of IgG1, IgG2, and IgG3 usually distribute in secondary MN, while many amounts of IgG4 are characteristic for IMN, indicating the fact that PLA_2_R antibody and THSD7A antibody are mostly of the IgG4 subclass.

#### 2.2.1 Phospholipase A_2_ Receptor Test for Diagnosis and Monitoring

Although the underlying mechanism of PLA_2_R in the pathogenesis of IMN is still unknown, the presence of anti-PLA_2_R antibodies is highly specific for IMN (Logt et al., 2021; Nieto-Gañán et al., 2021). A low occurrence of PLA_2_R antibodies was found in secondary MN (Porcelli et al., 2021), but in these patients, coincidental occurrence of IMN with related disease might be included. The level of PLA_2_R antibodies was detected in patients with other cause-induced nephrotic syndrome or healthy controls (Tomas et al., 2021). Several findings suggest that anti-PLA_2_R antibodies were associated with disease activity (Jurubită et al., 2021; Logt et al., 2021).

The commercial immunofluorescent test (Euroimmun, Lübeck, Germany) was applied to diagnosis. Anti-PLA_2_R antibodies occurred in serum of 52–86% of MN patients (Hofstra and Wetzels, 2012). Detections by using either Western blot or immunofluorescent approaches, and immunofluorescent tests have a lower sensitivity than the Western blot technique (Hoxha et al., 2017). The studies included patients with different ethnicity, long-standing disease, and treated in remission. It was also reported that initially antibodies against PLA_2_R lacked in serum of patients with PLA_2_R-related MN. An earlier study showed 10 patients with PLA_2_R antigen staining positive in immune complexes in the renal biopsy specimen from a cohort of 42 patients, with no measurable serum antibodies (Debiec and Ronco, 2011). Subsequent results showed that antibodies against PLA_2_R indeed were absent at disease the onset and could be detected during follow-up analysis (Ramachandran et al., 2015; van de Logt et al., 2015). We hypothesized that antibodies combined with antigen of podocytes with high affinity and only be detected when binding sites in renal tissues are saturated (van de Logt et al., 2015). Of note, the same research group also reported three patients with a high circulating concentration of antibodies against PLA_2_R who did not have detectable PLA_2_R in the glomeruli (Debiec and Ronco, 2011). This phenomenon attracts little attention. The immunofluorescent analysis only allowed semi-quantitative evaluation of antibodies against PLA_2_R by dilution steps. The enzyme-linked immunosorbent assay (ELISA) provided a precise quantitation for antibodies against PLA_2_R. A commercially available ELISA (Euroimmun) was introduced to clinical practice and widespread application (Dähnrich et al., 2013; Zhu et al., 2022). This ELISA determined total antibodies against PLA_2_R IgG. However, PLA_2_R antigen was observed in immune deposits in some patients, which suggested rapid clearance of antibodies and deposition in glomeruli. Conversely, determination of circulating antibodies was not always related to the presence of the antigen in the immune complex deposits, which indicated that not all antibodies to PLA_2_R are pathogenic. Evaluation of both circulating anti-PLA_2_R antibodies and PLA_2_R in kidney biopsy could better select patients for accurate therapy. In addition, anti-PLA_2_R antibodies showed a high diagnostic ability on IMN for the population with diabetic kidney disease (Wang et al., 2020a).

#### 2.2.2 Thrombospondin Type-1 Domain-Containing Protein 7A Test for Diagnosis and Monitoring

So far, THSD7A antibodies were not detected in healthy individuals or patients with other renal and systemic diseases (Tomas et al., 2014), presenting a 100% specificity for the damage of MN. However, another study has demonstrated that circulating autoantibodies against human podocyte antigen THSD7A was found in 5–10% of MN patients who did not present circulating anti-PLA_2_R autoantibodies (Tomas et al., 2014). The percentages of THSD7A-related IMN range from 3% to 9% in Europe and the United States. Notably, in large-scale THSD7A-associated MN patients, a tumor was found from diagnosis of MN (Hoxha et al., 2016). Of note, chemotherapy initiation caused a decreasing THSD7A antibody followed by a reduction of proteinuria (Hoxha et al., 2016). These findings showed that the immune system discerned cancer THSD7A as a foreign antigen mediating THSD7A antibody production, the latter binding to THSD7A on the surface of podocyte *in situ*.

#### 2.2.3 Combined Phospholipase A_2_ Receptor and Thrombospondin Type-1 Domain-Containing Protein 7A Test for Diagnosis and Monitoring

It is presently uncertain whether there are patients with dual PLA_2_R and THSD7A antibody positivity (Zhang et al., 2021a). When PLA_2_R antibodies were detected in serum and there is no evidence for secondary MN, one may consider that patients do not undergo a kidney biopsy sample, provided that patients presented normal or only mild renal function decline. In patients with kidney function injury, a biopsy could help us to exclude a crescentic form of MN or concurrence of other diseases and evaluate the chronic damage degree.

## 3 Treatment of Membranous Nephropathy

Currently, IMN patients are mainly treated with either calcineurin inhibitors, alkylating cytotoxic agents, B-cell depleting monoclonal antibody, or rituximab (Wang et al., 2018a; Hamilton et al., 2018; Yu et al., 2018). The use of calcineurin inhibitors led to a high relapse rate on their withdrawal. Alkylating cytotoxic agents were effective, but their use led to severely adverse effects. Rituximab-treated MN patients showed a decrease in anti-PLA_2_R antibody levels in the follow-up period (Ramachandran et al., 2018). However, it is worth noting that some natural products exhibit an excellent efficacy for MN treatment (Feng et al., 2020; Lang et al., 2020; Lu et al., 2021).

### 3.1 Immunosuppressive Treatment

Kidney Disease: Improving Global Outcomes guidelines recommend that corticosteroids and alkylating agents including cyclophosphamide or chlorambucil are prescribed for 6 months for MN patients with nephrotic syndrome after 6–12 months as a conservative treatment or with decreased baseline renal function (Stevens and Levin, 2013). However, these therapies showed some severe adverse effects. The severity of side effects had important drawbacks in this therapy. Therefore, the classical combined therapy should be optimized (Chen et al., 2020a). The latest results indicated that remission at first 3 months were higher in the steroid–tacrolimus group than in the steroid–cyclophosphamide group over an 18-month period (Zou et al., 2020). Although the incidence of adverse effects was not different between two groups, the incidence after first 3 months was decreased in the steroid–tacrolimus group. Levels of 24-h urinary protein and serum albumin improved in the steroid–tacrolimus group more than those in the steroid–cyclophosphamide group (Zou et al., 2020).

Hoxha et al. (2014)reported that antibody levels were significantly reduced after 3 months of immunosuppressive treatment by 81% and proteinuria by 39% in 133 MN patients with positive anti-PLA_2_R antibody. Patients with remission after 12 months presented decreased levels of anti-PLA_2_R antibodies at baseline compared to patients with no remission. Moreover, patients with high anti-PLA_2_R antibody concentrations achieved remission later than patients with low concentrations. The antibody concentrations remained increased in patients who did not achieve proteinuria remission.

Alternatively, decreasing proteinuria concentrations without an immunological response could not be interpreted as real remission. There is an increasing risk of relapse when treatment by frequently using cyclosporine is withdrawn. Persistence of the high anti-PLA_2_R antibody titer is a sign of ongoing immunologic activity that was associated with ongoing podocyte injury despite decreased proteinuria concentrations. Therefore, the efficacy of any immunosuppressive treatment need to be assessed based on immunologic remission induction (Bomback and Fervenza, 2018). Anti-PLA_2_R positivity reappearance and/or an elevation in the previously lower titer of anti-PLA_2_R antibody was a clear indicator of the impending relapse of MN, sometimes preceding the rise of proteinuria concentrations. Serial detection methods of anti-PLA_2_R antibodies during immunosuppressive treatment could improve personalized treatment of MN.

### 3.2 Rituximab

**FIGURE 3:**
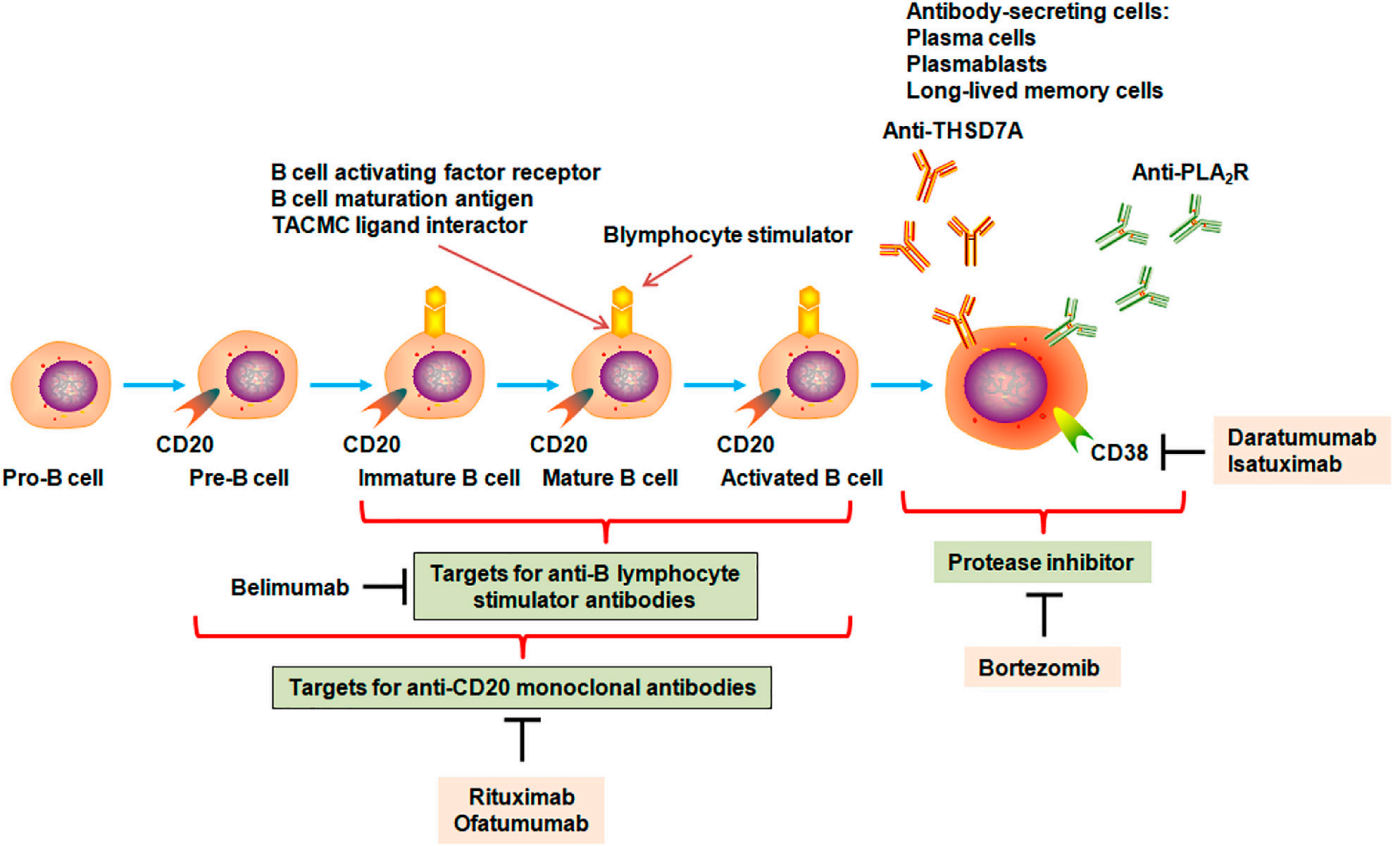
Targets for monoclonal antibodies in B cells for the MN therapy strategy. B cells originated from bone marrow stem cells as pro-B cells and mature to pre-B cells, immature B cells, mature B cells, and activated B cells, which could form plasma cells, plasmablasts, and long-lived memory plasma cells that could secrete antibodies such as immunoglobulin A, immunoglobulin E, immunoglobulin G, and immunoglobulin M. Autoreactive B-cell clones could generate anti-PLA_2_R and anti-THSD7A antibodies. When B cells became mature, they generated many biomarkers that were considered targets for monoclonal antibodies. Monoclonal antibodies rituximab and ofatumumab could bind and inhibit a number of CD20-positive B cells but not mature plasma cells, plasmablasts, and memory plasma cells that could not produce CD20 antigen. Plasma cells produced CD38 and could be a target for anti-CD38 antibodies by daratumumab and isatuximab. Immature, mature, and activated B cells produced receptors for B-lymphocyte stimulator including B-cell activating factor receptor, B-cell maturation antigen, transmembrane activator, and calcium-modulating cyclophilin (TACMC) ligand interactor. Belimumab could inhibit B-cell differentiation into plasma cells by suppressing interaction between B-lymphocyte stimulator and its receptors. Bortezomib, a proteasome inhibitor, could inhibit antibody production by mediating plasma cell apoptosis, while anti-CD38 antibodies daratumumab and isatuximab could directly mediate plasma cell cytolysis.

After nearly 40 years of empirical treatment, the identification of newer anti-PLA_2_R and anti-THSD7A autoantibodies was an unprecedented breakthrough for the understanding of underlying pathophysiological mechanisms of MN and its interventions specifically aimed at preventing antibody production (Figure 3). Rituximab was one of the first-line drugs for the treatment of moderate and high-risk IMN. The latest study demonstrated that 91 patients treated by rituximab achieved anti-PLA_2_R antibody depletion at 6 months; 58.2% of patients showed clinical remission at 12 months (Gao et al., 2021). Further analysis indicated that high proteinuria levels and persistent positive anti-PLA_2_R antibodies could be independent risk factors for no remission. The remission rate treated using rituximab as an initial regimen was increased compared to rituximab as an alternative regimen. In addition, 45 adverse events occurred in 37 patients. Rituximab effect was determined for the patients with PLA_2_R-associated MN and stage four or five chronic kidney disease (CKD) (Hanset et al., 2020). In total, 10 treatment courses caused an increase in an estimated glomerular filtration rate and remission of nephrotic syndrome. In contrast, four patients treated by rituximab were unsuccessful and required chronic hemodialysis within 1 year. Immunological remission was observed after 11 treatments and was related to response. However, three patients showed severe adverse events (Hanset et al., 2020). These findings suggest that rituximab showed effective and reasonably safe in PLA_2_R-related MN with stage four or five CKD. Immunological remission is related to a beneficial clinical outcome.

Prior to the finding that anti-PLA_2_R autoantibodies affected MN pathogenesis, *in vivo* animal experiments had consistently demonstrated that antibodies generated by autoreactive B-cell clones triggered events that led to glomerular barrier damage and proteinuria development (Brglez et al., 2020). Consistent with these findings, cyclophosphamide showed an inhibitory effect on production of B-cell antibody in MN, adding to the non-specific antimitotic and immunosuppressive nature that induced some side effects. A monoclonal antibody against B-cell surface antigen CD20 has performed whether targeted B-cell depletion with inhibition of autoantibody production ameliorated MN patients while reduced side effects of immunosuppressants and steroids. Therefore, rituximab, as an anti-CD20 monoclonal antibody (Figure 3), was used in MN patients. MN patients treated by rituximab showed reduced levels of circulating anti-PLA_2_R antibody and proteinuria within several months (Gao et al., 2021; Gauckler et al., 2021). Ruggenenti et al. (2015) demonstrated that rituximab-treated patients with IMN showed a partial or complete remission, whereas anti-PLA_2_R antibody-positive patients showed a remission demonstrated by a decrease in antibody titer before treatment. These patients presented complete antibody depletion 6 months after the beginning of rituximab regimen. The finding from all patients showed that depletion of anti-PLA_2_R antibodies preceded complete remission. Early decreasing of anti-PLA_2_R antibody titer by 50% was in line with a reduction in proteinuria levels by 50% by 10 months, whereas rituximab was not associated with polymorphisms of PLA_2_R1.

Recently, it was reported that early response to either cyclosporine or rituximab was complete, and partial remission was observed in 130 patients treated by cyclosporine or rituximab, which showed similar results. However, partial or complete remission was faster in rituximab-treated patients than cyclosporine-treated patients at 24 months Fervenza et al. (2019). However, cyclosporine was withdrawn at 12 months, which reflected the risk of early relapse after cyclosporine withdrawal. Of note, the reduction of anti-PLA_2_R antibodies was faster in rituximab-treated patients than the cyclosporine-treated patients (Fervenza et al., 2019). These findings illuminated that immunological remission by depleting anti-PLA_2_R antibodies was promising for clinical remission.

Recently, a randomized and open-label controlled experiment was performed on 86 patients with IMN and nephrotic syndrome and assigned 43 each to receive 6-month cyclical intervention with the corticosteroid–cyclophosphamide group or sequential intervention with the tacrolimus–rituximab group (Fernández-Juárez et al., 2021). The results showed 83.7% of patients treated by the corticosteroid–cyclophosphamide group and 51.8% of patients treated by the tacrolimus–rituximab group exhibited complete or partial remission at 24 months. Complete remission occurred in 60% of patients treated by the corticosteroid–cyclophosphamide group and 26% of patients treated by the tacrolimus–rituximab group (Fernández-Juárez et al., 2021). Anti-PLA_2_R titers were significantly decreased in both groups, but the proportion of anti-PLA_2_R-positive patients who showed anti-PLA_2_R antibody depletion was higher at 3 and 6 months in the corticosteroid–cyclophosphamide group than the tacrolimus–rituximab group (Fernández-Juárez et al., 2021). Severe adverse effects were similar in both groups. Therefore, the corticosteroid–cyclophosphamide treated remission in many patients with IMN compared to tacrolimus–rituximab. In addition, Tian et al. (2022)assessed the efficacy and safety of tacrolimus combined with corticosteroids in patients with IMN and reported that 75 patients with renal biopsy MN and nephrotic syndrome were treated by rituximab or non-immunosuppressant. The depletion of anti-PLA_2_R was demonstrated at 6 months in 50% of patients intervened by rituximab and only in 12% of patients intervened by non-immunosuppressant Dahan et al. (2017).

### 3.3 Pharmacological Effects of Natural Products on Membranous Nephropathy

Natural products or traditional Chinese medicines have been long used in patients and considered an alternative therapeutic strategy for prevention and treatment of glomerular-associated diseases including MN (Feng et al., 2020), glomerulonephritis (Gianassi et al., 2019; Wang et al., 2021), diabetes (Chen et al., 2020b; Wang et al., 2020b; Fang et al., 2021; Su et al., 2021; He et al., 2022), and diabetic nephropathy (Li et al., 2020a; Yang et al., 2020; Wang, 2021; Xuan et al., 2021; Yang and Wu, 2021; Zhou et al., 2021; Liu et al., 2022). Earlier finding have demonstrated that a 77-year-old woman with IMN was treated by *Astragalus membranaceus* and achieved clinical remission without using immunosuppressive therapy (Ahmed et al., 2007). A multicenter randomized controlled clinical study assessed efficacy and safety of Shenqi particle for patients with IMN. Shenqi particle showed a beneficial effect on patients with IMN and nephrotic syndrome (Chen et al., 2013). In addition, Jian Pi Qu Shi formula treatment showed improvement in 15 patients, who failed to respond immunosuppressive therapy, and showed that 80% of the patients achieved clinical remission, whereas no obvious adverse effects were observed after 1-year follow-up (Shi et al., 2018). Similarly, Shulifenxiao formula as a clinical cocktail therapy also showed a beneficial intervention effect on steroid and immunosuppressant-resistant refractory IMN patients (Cui et al., 2021). Therefore, these formulas might be an alternative therapy for steroid and general immunosuppressant-resistant IMN patients. The combination of *Tripterygium wilfordii* multi-glycosides and prednisone is considered an effective therapy for IMN. The remission probability was similar for both *Tripterygium wilfordii* multi-glycosides and tacrolimus (Jin et al., 2020). Traditional Chinese medicines also could improve immunosuppressant efficacy. Wuzhi capsule could increase blood FK506 concentration in patients with IMN (Zhang et al., 2019). These studies have indicated that traditional Chinese medicines can effectively improve IMN and reduce proteinuria, but the underlying mechanism is still elusive.

Mechanistically, Wu et al. recently reported that 24-h urine protein level was significantly decreased, and kidney histological injury was restored in the CBSA-induced rats treated by Wenyang Lishui decoction. Similarly, an *in vitro* experiment showed that the apoptosis rate was increased in CBSA-induced mouse podocytes, while it was decreased when treated by Wenyang Lishui decoction, which was associated with downregulation of p53 mRNA and protein expression and upregulation of Bcl-2 mRNA and protein expression (Lu et al., 2020). An earlier study has revealed that Astragaloside IV might ameliorate complement attack complex-mediated podocyte lesion *via* inhibiting extracellular-regulated protein kinase expression (Zheng et al., 2012) (Figure 2). Recently, Tian et al. (2019)revealed that administration of Sanqi oral solution mitigated MN by lowering proteinuria, increasing serum albumin, and retarding renal damages in the experimental rat model of MN induced by CBSA. Sanqi oral solution also inhibited depositions of C3 and IgG and restored the protein expressions of podocin and synaptopodin, which were associated with the nuclear factor-κB (NF-κB) signaling pathway (Tian et al., 2019). The NF-κB signaling pathway plays an important role in immune modulation. Recent studies revealed that the NF-κB pathway was involved in the pathogenesis of MN (Sutariya et al., 2017). Liu et al. (2019)demonstrated that Zhenwu decoction reduced urine protein levels and alleviated kidney damage in the rat model of MN; furthermore, treatment with Zhenwu decoction could downregulate the protein expressions of IgG, C3, and desmin as well as upregulate podocin expression in glomerulus. The same research group demonstrated that Zhenwu decoction inhibited the advanced glycation end by suppressing the expression of receptor for advanced glycation end products in podocyte, which reduced oxidative stress in podocyte (Wu et al., 2016). These findings revealed that natural products ameliorate MN through targeting inflammation. However, MN is a non-inflammatory autoimmune disease of the kidney glomerulus. Taken together, the underlying mechanism should be investigated in the future.

In addition, Yu et al. (2020) reported that Chinese herbal injections were demonstrated to be superior to treatment of chemical drugs alone in the treatment of primary nephrotic syndrome and might be beneficial for patients with primary nephrotic syndrome. The combination of chemical drugs and Yinxingdamo injection and chemical drugs and Danhong injection had the potential to be the best Chinese herbal injections relative to total clinical effectiveness, serum albumin, and 24-h urinary protein excretion. Moreover, Li et al. (2020b) and Li et al. (2020c) demonstrated that Zhen-Wu-Tang could ameliorate immunoglobulin A nephropathy in rats.

### 3.4 Future Therapy and Directions

The integrated assessment of the levels of autoantibody and albumin in serum and proteinuria in patients could provide MN diagnosis and individually tailored treatment protocols. Traditional, toxic, and non-specific immunosuppressant will be replaced by safe and disease-specific agents, such as B-cell-targeting anti-CD20 antibodies (Figure 3), providing a new treatment paradigm based on the principles of precision medicine and personalized therapy. Although great advances were achieved in MN pathogenesis, a number of critical issues remain unsolved. For example, how the immune response is triggered and spreads remains unknown; the conditions that resulted in the appearance of PLA_2_R epitope of podocytes and the events that mediated immunization are elusive; the aim of antigen-driven therapy has still not been fulfilled. Although anti-PLA_2_R and anti-THSD7A antibodies as the leading diagnosis approaches were extensively applied to MN patients, some questions including autoimmune response, antigenic epitopes, and podocyte injury-associated with signaling pathways remain unresolved. In addition, when the patients have renal biopsy of MN, the clinicians need to answer two key issues. If patients are PLA_2_R-positive, then what are the risk factors of progression to renal failure? If patients are PLA_2_R-negative, then the causes of disease development and progression are primary or secondary?

Owing to the adverse effects of currently available immunosuppressive agents, a better understanding of MN pathomechanisms will provide more specific concept-directed therapy strategies. Based on the critical role of IgG antibodies in MN, targeting B lymphocytes by anti-CD20 antibody might be a specific and effective therapy to ameliorate MN by blocking antibody formation (Figure 3). However, responses are wide, and further research is needed to identify those patients who are benefitted by rituximab treatment and those who are non-effective. A clinical study showed that adrenocorticotropic hormone exerted its role through activated melanocortin receptors (MC1R–MC5R) that existed in the whole body were effective therapy for IMN patients who failed immunosuppressive treatment (Markell et al., 2019; Wu et al., 2021b). Melanocortin one receptor occurred in B cells, T cells, podocytes, and antigen-presenting cells. Melanocortin one receptor agonists could lower proteinuria, improve glomerular morphology, and retard oxidative stress in passive Heymann nephritis rats, which were associated with decrease in production of nephritogenic antibodies, direct targeting effect on podocytes, and stabilizing glomerular architecture. Further experiment should be performed to adrenocorticotropic hormone effect. The pathophysiological mechanisms have an important effect on patient care, including kidney biopsy, diagnosis, monitoring, and therapy.

Intriguingly, natural products are one of the most promising therapies for a broad spectrum of refractory diseases including coronavirus disease 2019 and its complications (Chen et al., 2018a; Chen et al., 2018b; Izzo et al., 2020; Yang et al., 2021a; Yang et al., 2021b; Zhang et al., 2021b; Singla et al., 2021; Zuo et al., 2021). Mounting natural products have been demonstrated to exhibit excellent efficacy for CKD treatment (Wang et al., 2018b; Zhang et al., 2020c; Geng et al., 2020; Meng et al., 2020; Miao et al., 2020; Lan, 2021; Li et al., 2021; Luo et al., 2021; Miao et al., 2022; Yu et al., 2022). Although a number of natural products could mitigate MN, the natural products in MN application are still in its infancy compared with CKD. Therefore, whether natural products can abolish MN, for which the study should be carried out on the animal models and patients with MN in the future.

## Data Availability

The raw data supporting the conclusion of this article will be made available by the authors, without undue reservation.

## Abbreviation

CBSA, cationic bovine serum albumin; CKD, chronic kidney disease; ELISA, enzyme-linked immunosorbent assay; GBM, glomerular basement membrane; IMN, idiopathic membranous nephropathy; MN, membranous nephropathy; NEP, neutral endopeptidase; NF-κB, nuclear factor-κB; PLA_2_R, M-type phospholipase A_2_ receptor; THSD7A, thrombospondin type-1 domain-containing protein 7A.
